# Elevated expression of both mRNA and protein levels of IL-17A in sputum of stable Cystic Fibrosis patients

**DOI:** 10.1186/1465-9921-11-177

**Published:** 2010-12-10

**Authors:** Ann Decraene, Anna Willems-Widyastuti, Ahmad Kasran, Kris De Boeck, Dominique M Bullens, Lieven J Dupont

**Affiliations:** 1 Laboratory of Pneumology, KULeuven, Herestraat, Leuven, Belgium; 2 Clinical Immunology, KULeuven, Herestraat, Leuven, Belgium; 3 Department of Pediatrics, University Hospital Gasthuisberg, Herestraat, Leuven, Belgium; 4 Department of Pneumology, University Hospital Gasthuisberg, Herestraat, Leuven, Belgium

## Abstract

**Background:**

T helper 17 (Th17) cells can recruit neutrophils to inflammatory sites through production of IL-17, which induces chemokine release. IL-23 is an important inducer of IL-17 and IL-22 production. Our aim was to study the role of Th17 cells in cystic fibrosis (CF) lung disease by measuring IL-17 protein and mRNA levels and IL-22 and IL-23 mRNA in sputum of clinically stable CF patients and by comparing these levels with healthy controls.

**Methods:**

Sputum induction was performed in adult CF patients outside of an exacerbation and healthy control subjects. IL-17A protein levels were measured in supernatants with cytometric bead array (CBA) and RNA was isolated and quantitative RT-PCR was performed for IL-17A, IL-22 and IL-23.

**Results:**

We found significantly higher levels of IL-17A protein and mRNA levels (both: p < 0.0001) and IL-23 mRNA levels (p < 0.0001) in the sputum of CF group as compared to controls. We found very low levels of IL-22 mRNA in the CF group. The levels of IL-17 and IL-23 mRNA were higher in patients chronically infected with *Pseudomonas aeruginosa *(*P. aeruginosa*) as compared to those who were not chronically infected with *P. aeruginosa*. The presence of *Staphylococcus aureus *(*S. aureus*) on sputum did not affect the IL-17 or IL-23 levels. There was no correlation between IL-17 or IL-23 levels and FEV_1 _nor sputum neutrophilia.

**Conclusion:**

The elevated levels of IL-17 and IL-23 might indicate that Th17 cells are implicated in the persistent neutrophil infiltration in CF lung disease and chronic infection with *P. aeruginosa*.

## Background

The major cause of morbidity and mortality in cystic fibrosis (CF) is lung damage characterized by bronchiectasis. This damage is the result of the vicious cycle of chronic infection and inflammation with production of harmful products such as proteases and oxidants secreted mainly by neutrophils. A major factor in the respiratory health of CF subjects is chronic *Pseudomonas aeruginosa *(*P. aeruginosa*) infection which is associated with a poor clinical outcome [1].

The role of the innate immunity in the pathophysiology of CF lung inflammation with a dominant neutrophilic type of inflammation has been established [2]. The role of the cellular, adaptive immunity however remains unclear but there is some evidence that lymphocytes might be involved. Aggregates of T and B lymphocytes were found beneath the epithelial layer in lung parenchyma of transplanted CF patients [3]. Histological analysis of bronchial biopsies of CF patients with chronic stable disease shows that lymphocytes are scattered throughout the subepithelium [4]. In healthy individuals, T cells express the cystic fibrosis transmembrane conductance regulator (CFTR) and defective CFTR protein affects the cytokine production by these T cells [5]. Also, the role of Th1 and Th2 cells and expression of their cytokines has been investigated in cystic fibrosis lung inflammation [6].

Antigen presenting cells such as dendritic cells (DC) that are activated by bacterial antigens in the bronchial mucus layer produce interleukin-23 (IL-23) [7], a pro-inflammatory cytokine. T helper 17 (Th17) cells produce IL-17A upon binding of IL-23 to its receptor on the T cell membrane. IL-17A is a pro-inflammatory cytokine of the IL-17 family that is mainly produced by Th17 cells [8]. The role of this newly discovered T helper subset in pulmonary inflammation has been described in numerous inflammatory diseases such as asthma [9] and chronic rejection after lung transplantation [10]. IL-17A induces granulopoiesis via induction of granulocyte colony-stimulating factor (G-CSF) and neutrophil recruitment via induction of chemotactic mediators such as IL-8 [11]. IL-22 is another cytokine that, in addition to IL-17, is produced by the Th17 lineage [12]. Both IL-22 and IL-17A have been shown to be crucial for maintaining local control of the Gram-negative pulmonary pathogen, *Klebsiella pneumoniae *in a mice model of lung infection [13].

The role of the IL-17A/IL-23 axis and Th17 cells in cystic fibrosis remains unclear. McAllister et al. [14] found elevated IL-17A and IL-23 protein levels in bronchoalveolar lavage (BAL) fluid and sputum of 8 CF patients during exacerbation. We hypothesized that IL-17A might be partly responsible for neutrophilic inflammation in the airways, and that there is chronic activation of the IL-23/IL-17A axis in CF airways in "stable" conditions (without exacerbation or intravenous (IV) antibiotic therapy).

The aims of this study were to quantify both protein and mRNA levels of IL-17A and mRNA levels of IL-22 and IL-23 in sputum of stable CF patients and to relate expression of these cytokines in sputum to the chronicity of airway infection.

## Methods

### Study population and study design

Adult (≥16 years) CF patients who did not have an exacerbation (defined by increase in symptoms, a deterioration of the FEV_1 _and/or documented radiological changes) and who were not on IV antibiotic therapy for at least 8 weeks, were recruited at the adult CF outpatient clinic of the university hospital Gasthuisberg. Healthy controls were recruited among students and research fellows of the KULeuven who had no history of respiratory diseases (no asthma and no symptomatic allergies). Informed consent was obtained from all subjects and the study was approved by the local ethical committee.

Clinical characteristics of CF patients were obtained from the hospital records. Spirometry was performed at the time of sputum induction and compared to spirometry results during previous hospital visits. The *P. aeruginosa *infection status was determined and patients were categorized as never, free, intermittent or chronic using the Leeds criteria [15] and specific anti-Pseudomonas IgG antibody levels as described previously [16].

Patients were considered to be "never infected" when *P. aeruginosa *was never cultured from sputum. Patients were "free from infection" when no *P. aeruginosa *was grown from the cultures during the previous 12 months, but when patients had been positive before this period. "Intermittent infection" meant that 50% of sputum samples were positive for *P. aeruginosa *(with a minimum of 4 samples in different months over one year) and patients were defined as "chronically infected" when more than 50% of the monthly samples were positive and with *P. aeruginosa *IgG antibody levels ≥ 17 AU. A similar approach was used to categorize *S. aureus *infection.

### Sputum induction

Sputum induction in healthy controls was performed by inhaling increasing concentrations of hypertonic saline (3% - 4% - 5%) and using a DeVilbiss nebuliser (Ultra-Neb 2000 model 200HI) [17]. Each concentration was inhaled for 7 minutes and subjects were asked to expectorate sputum after each inhalation period. Sputum induction in CF patients was performed with the same nebuliser, but with the use of a hypertonic saline solution of 5% NaCl only during 10-20 minutes until enough sputum was collected. Pre-treatment with a bronchodilator (salbutamol) was done in both healthy control subjects and CF patients.

### Sputum processing

Sputum samples were processed by selecting mucus plugs to avoid salivary contamination. Sputum processing was performed with an adapted protocol from Pizzichini et al. [18] as previously described [17]. In brief, the selected sputum plugs were incubated in a volume of 4 times the weight of the plugs of Hank's balanced salt solution (HBSS) (BioWitthaker Europe, Cambrex, Verviers, Belgium) with 3% bovine serum albumin (BSA) and 0.1% dithiothreitol (DTT) (Sigma, St. Diego, USA) during 15 minutes on a bench rocker. The cells were washed with 4 times the weight of the plugs with Dulbecco's phosphate solution (D-PBS) (BioWitthaker Europe, Cambrex) with 0.5% BSA during 5 minutes on a bench rocker. Samples were filtrated through a 70 μm Falcon cell strainer (BD Biosciences, San Jose, California, USA) and centrifuged at 1500 rpm during 10 minutes. The supernatant of each sample was kept at -20°C for protein analysis. A small part of the cell pellet was used for cytospins (see further), the rest was frozen at -80°C with RLT lysis buffer (Qiagen, Maryland, USA) for further RNA isolation.

### Cell counts

Cytospins of sputum cells were made in a Shandon cytocentrifuge (Techgen, Zellik, Belgium) and stained with May-Grünwald Giemsa (Diff-Quick stain kit, Medion Diagnostics, Düdingen, Switzerland). Per slide, 250 leukocytes and epithelial cells were counted and percentages of inflammatory cells (macrophages, neutrophils, eosinophils and lymphocytes) were calculated on the total number of leukocytes without considering the epithelial cells.

Absolute numbers of each cell type were calculated by multiplying the percentages on cytospins with the total cell count number counted after isolation.

### mRNA measurements in sputum samples

RNA was isolated using the RNeasy mini kit (Qiagen). cDNA synthesis was performed with Ready-To-Go You-Prime First-Strand Beads (GE Healthcare Life Sciences, Uppsala, Sweden). RT-PCR was performed in an ABI prism 7700 Sequence Detector System (Applied Biosystems, Foster, USA) for IL-17A, IL-22 and IL-23 with specific Taqman probes and primers, and using PCR Reaction mix (Invitrogen, Merelbeke, Belgium). IL-22 primers and probe were developed in the laboratory of clinical immunology using Primer Express (Applied Biosystems) (sequences: forward primer: 5' ttc atg ctg gct aag gag gc 3'; reverse primer: 5' gca gcg ctc act cat act gac t; Taqman probe: 5' TAM agc ttg gct gat aac aac aca gac gtt cgt TAMRA 3'). Primers and probes for IL-17A and IL-23 were previously published (IL-17A [9]; IL-23 [10]). cDNA plasmids expressing linear amounts of the target gene, were used as a standard. 18S rRNA was used as housekeeping gene (Pre-Developed TaqMan^® ^Assay Reagent Control kit, Applied Biosystems) and all mRNA values were normalized to 18S rRNA expression by using ratio of the number of copies of the cytokine and the housekeeping gene RNA multiplied by 10^4^.

### Protein measurements in sputum samples

The BD™ CBA Flex Set System and buffer master kit (BD Biosciences) was used to measure protein levels of IL-17A in the sputum supernatants. Measurements were performed as instructed by the manufacturer.

We did previous protein measurements on these samples including ELISA from different companies and under different conditions, but we failed to measure IL-17A. This was probably due to technical reasons, inherent to the determination of cytokine protein levels in induced sputum supernatants including the use of DTT to process the samples and the dilution factor. Therefore, we could not perform the CBA in all the samples. We only had a very limited amount of control samples left so we included new control samples. We opted to use CBA, since this is a more sensitive technique than ELISA, hence the higher concentrations of other inflammatory markers (e.g. IL-8) measured in CBA compared to ELISA in previous studies [19].

### Statistical analysis

For the statistical analysis of the results, Graphpad Prism (Graphad software Inc, San Diego, USA) was used. For the comparison of two groups, a non-parametric Mann-Whitney U test was used. Chi-square test was used to compare categorical variables. Correlations were checked with a non-parametric Spearman test. Normality was checked with a Kolgomorov-Smirnov test and variances were tested with F-test. A p-value < 0.05 was considered as significant for all tests.

## Results

### Study population

Thirty eight stable adult CF patients and 11 healthy controls were included in the study. Characteristics of CF patients and control subjects are shown in Table 1. Twenty-two of the CF patients were using inhaled antibiotics as maintenance treatment. 21 patients of the total group were using azithromycin. The never/free/intermittent group consisted of 17 patients and the chronic *P. aeruginosa *group consisted of 21 patients. This second group tended to have a lower FEV_1 _although this difference was not statistically significant (p = 0.096 for FEV_1 _at time of sputum induction).

**Table 1:** Characteristics of healthy controls and CF patients, divided in subgroups according to P. aeruginosa infection status

	CF never/free/intermittent *P. aeruginosa*	CF chronic *P. aeruginosa*	control subjects
Nr. of subjects	17	21	11
Age (mean ± SD) in years	26.6 ± 8.0	25.1 ± 4.3	27.5 ± 10.9
Gender (F/M)	11/6	9/12	10/1
BMI (mean ± SD) in kg/m^2^	21.6 ± 3.0	20.2 ± 2.5	NA
Genotype (# of pts dF508del homozygous)	2	16*	NA
Exocrine pancreas insufficiency (%)	64.7	100**	NA
Current FEV_1 _(% predicted) (mean ± SD)	72.8 ± 17	61.9 ± 17.4	Normal values
Mean FEV_1 _previous year (% predicted) (mean ± SD)	73.8 ± 16.6	65.1 ± 16.3	NA

SD: standard deviation, F: female, M: male, NA: not available, pts: patients.

*: p < 0.0001 (Chi-square test), **: p = 0.003 (Chi-square test).

### Differential cell counts

Nine out of 11 cytospins from the controls and 34 out of 38 from the CF patients were of good quality to allow differential cell counts. Percentages and absolute numbers of each inflammatory cell type are given in Table 2. In the CF group we could observe a dominant neutrophilia compared to the healthy controls.

**Table 2:** Total and differential cell counts

Cell counts (mean ± SD)	CF never/free/intermittent *P. aeruginosa*	CF chronic *P. aeruginosa*	control subjects
Total cell count (10^6^)	3.8 ± 2.9	4.1 ± 4.2	0.7 ± 0.4
% of neutrophils	89.3 ± 11.5	93.3 ± 4.0	41.4 ± 12.4
Absolute numbers of neutrophils (10^6^)	3.4 ± 2.8	4.0 ± 3.8	0.32 ± 0.28
% of lymphocytes	0.4 ± 0.6	0.7 ± 1.0	0.8 ± 0.3
Absolute numbers of lymphocytes (10^4^)	1.7 ± 2.5	1.8 ± 4.6	1.7 ± 1.6
% of macrophages	9.2 ± 11.2	5.5 ± 3.1	57.3 ± 12.3
Absolute numbers of macrophages (10^5^)	3.0 ± 4.1	2.8 ± 4.6	3.8 ± 1.6
% of eosinophils	1.1 ± 2.6	0.5 ± 0.6	0.4 ± 0.05
Absolute numbers of eosinophils (10^4^)	3.1 ± 8.3	2.1 ± 4.5	0.15 ± 0.36

Absolute numbers of cells per milliliter and percentages on total leukocytes of sputum cells in healthy controls and patients with CF patients, divided in subgroups according to *P. aeruginosa *infection status and healthy controls.

### Cytokine levels in sputum

**Figure 1:**
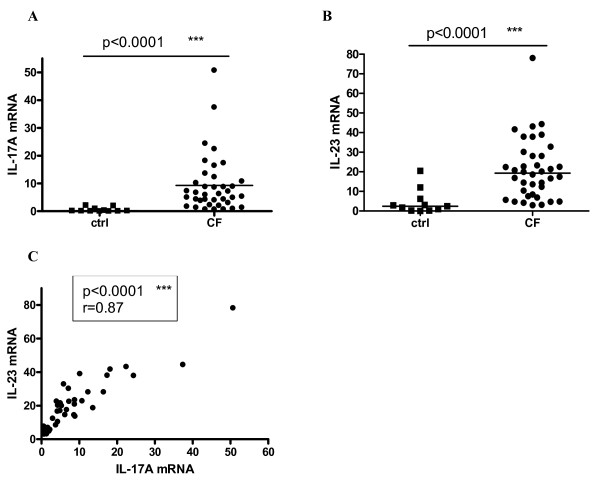
**IL-17A and IL-23 mRNA levels: CF group compared to controls**. IL-17A **(A) **and IL-23 **(B) **mRNA levels in controls (n = 11) compared to CF group (n = 38). mRNA levels were measured by RT-PCR. Values were normalized to 18S rRNA (ratio multiplied by 10^4^). Comparison of controls and CF group was done by nonparametric Mann-Whitney U test (p < 0.05 significant). Median levels are shown by the line. **(C)**: Correlation between IL-17A and IL-23 mRNA in the CF group (n = 38). Spearman correlation test was used (p < 0.05 significant) (r = correlation coefficient).

We measured mRNA levels by RT-PCR in all healthy controls (n = 11) and CF patients (n = 38). Median (P25-P75) IL-17A mRNA levels were 0.14 (0.06-0.95) for controls and 6.1 (2.6-11.6) for CF and median IL-23 mRNA levels 2.4 (0.25-6.2) for controls and 19.3 (8.0-29.2) for CF (p < 0.0001 for both; figure 1A and 1B). IL-22 mRNA levels were negative or very low in the CF group (data not shown). In the CF group, there was a strong correlation between IL-17A mRNA and IL-23 mRNA levels (r = 0.87; p < 0.0001) (Figure 1C). In healthy controls this correlation was not as strong, but still significant (r = 0.62; p = 0.043) (data not shown).

**Figure 2:**
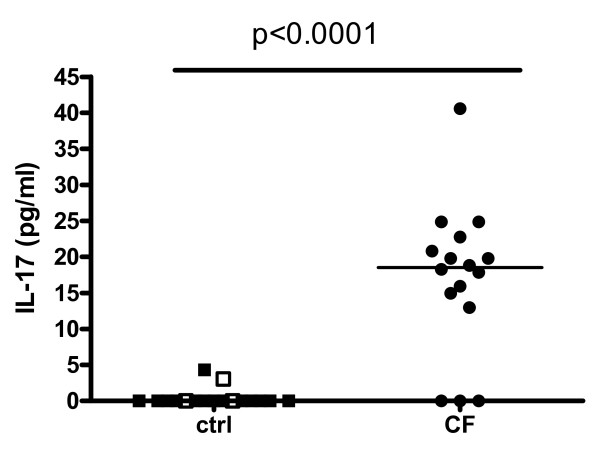
**IL-17A protein levels: CF group compared to controls**. IL-17A protein levels in controls (n = 17) compared to CF group (n = 16). Protein levels were measured by CBA and multiplied by 9 to correct for dilution into sputum buffers. Open symbols in control group are original 3 controls and closed symbols are new controls. Comparison of controls and CF group was done by nonparametric Mann-Whitney U test (p < 0.05 significant). Median levels are shown by the line.

We measured IL-17A protein levels with CBA. We obtained additional sputum supernatant samples in 14 different control subjects (8 female; mean age 27.3) next to the 3 control samples left from the original group. In the 16 samples left from the original CF patient group, we found median IL-17A levels of 18.54 (13.46-22.28) pg/ml with only 3 samples below the detection limit, but in the control group all values fell below detection limit (0.3 pg/ml undiluted). Thus, IL-17A protein levels in CF patients were significantly higher compared to IL-17A protein levels in the total group of controls (p < 0.0001; Figure 2).

There was no difference in IL-17A/IL-23 expression between patients with or without oral azithromycin therapy, also when we only looked within the group of chronically infected patients (data not shown). The group taking inhaled antibiotics tended to have higher levels of IL-17A mRNA (p = 0.054).

### IL-17A and IL-23 expression and infection status and disease severity

**Figure 3:**
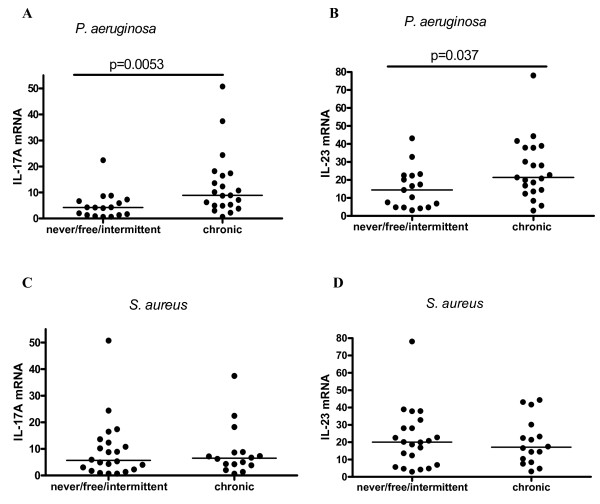
**Comparison of IL-17A and IL-23 levels between patients with different colonisation status of *P. aeruginosa *and *S. aureus***. Comparison of IL-17A and IL-23 mRNA levels between patients with different colonisation state of *P. aeruginosa *(A and B) (never/free/intermittent: n = 17 and chronic: n = 21) and *S. aureus *(never/free/intermittent: n = 22 and chronic: n = 16) (C and D) according to the Leeds criteria. mRNA values were normalized to 18S rRNA (ratio multiplied by 10^4^). Comparison between the groups was done by nonparametric Mann-Whitney U test (p < 0.05 significant). Median levels are shown by the line.

IL-17A mRNA and IL-23 mRNA levels were significantly higher in the chronic *P. aeruginosa *group as compared to the never/free/intermittent group (p = 0.0053 and p = 0.037 respectively; Figure 3A and 3B).

IL-17A protein levels were available in 16 CF patients (8 in *P. aeruginosa *never/free/intermittent group and 8 in chronic group). Median concentration of IL-17A was 19.04 (13.46-22.03) and 18.32 (3.98-23.83) pg/ml in chronic *P. aeruginosa *group and in never/free/intermittent group, respectively. These values were not statistically different.

There was no correlation between IL-17A nor IL-23 levels and the anti-Pseudomonas IgG antibody levels (data not shown).

There was no difference in IL-17A and IL-23 mRNA levels between the patients chronically infected with *S. aureus *as compared with the never/free/intermittent group (p = NS; Figure 3C and 3D). The difference was also not significant when we excluded the chronic *P. aeruginosa *patients (data not shown). There was no difference in IL-17A protein levels amongst the different groups of *S. aureus *infection (data not shown).

There was no correlation between IL-17A protein and mRNA nor IL-23 mRNA levels and FEV_1 _% predicted at time of sputum induction nor with mean FEV_1 _% of previous year nor with sputum neutrophil counts (expressed either as percentage or absolute number) (data not shown).

## Discussion

In the present study, we found elevated levels of both IL-17A protein and mRNA levels and also of IL-23 mRNA in sputum of clinically stable CF patients as compared to healthy controls, thus suggesting a potential role of Th17 cells in the pathophysiology of CF lung disease.

These results were confirmed by significantly increased IL-17A protein levels and in line with previous data on IL-17A expression in CF patients during exacerbation. McAllister et al [14] found elevated IL-17A protein levels in sputum of adult CF patients during a pulmonary exacerbation and in these patients, there was a decrease of the IL-17A protein levels after IV antibiotic therapy. The same authors found elevated IL-17A protein levels in BAL fluid of children with CF also during a pulmonary exacerbation [20]. Our data suggest a chronic activation of the IL-23/IL-17A axis in CF airways even outside an episode of pulmonary exacerbation.

Our results indicate that infection with *P. aeruginosa *may promote the IL-17A and IL-23 expression in CF, since we found that patients chronically infected with *P. aeruginosa *have higher mRNA expression of both cytokines than patients who were not chronically infected. IL-17A protein levels were not different between *P. aeruginosa *subgroups but this may be due to the limited number of sputum samples available for protein measurement as explained in the methods section.

Previous studies in animal models have shown that *P. aeruginosa *airway infection is able to induce IL-23 release in wild-type C57BL/6 mice while in IL-23 knock-out mice, the airway inflammation caused by *P. aeruginosa *was significantly reduced [21]. These data suggest that the continuous presence of Pseudomonal antigens in the mucus layer may feature as a strong stimulus for DC activation followed by IL-23/IL-17A production.

Our results did not show a relation between IL-17A mRNA and IL-23 mRNA levels and the *S. aureus *infection status. In the subgroup of 6 patients infected with both *P. aeruginosa *and *S. aureus*, IL-17A mRNA and IL-23 mRNA levels were not significantly higher than in those chronically infected with *P. aeruginosa *alone. This is in contrast with findings of Sagel et al., who demonstrated that the presence of both *P. aeruginosa *and *S. aureus *had an additive effect on concentrations of inflammatory markers in BAL [22]. Bodini et al. showed that leukotriene B4 (LTB4) and IL-8 levels in exhaled breath condensate were also related to the type of bacterial infection in CF patients, with highest levels in CF patients infected by *P. aeruginosa *[23]. *P. aeruginosa *might stimulate the IL-17A/IL-23 axis in the airways via activation of Toll like receptor (TLR) 4, similar to gram negative *Klebsiella pneumonia *[24], while peptidoglycans and bacterial lipoproteins from Gram-positive bacteria such as *S. aureus *mediate their response trough TLR2. Whether stimulation of *P. aeruginosa *and *S. aureus *via different TLR pathways may result in a different IL-23 expression and different activation of DC and ultimately in a different disease severity in CF remains speculative.

In our study, CF patients with chronic *P. aeruginosa *infection had a trend towards a lower FEV_1_. Although this difference was not significant, this trend suggests a more severe pulmonary disease in CF patients with *P. aeruginosa *and is consistent with previous findings [16,25].

Our results did not show a significant correlation between IL-17A or IL-23 mRNA levels and lung function parameters. We hypothesize that the lack of correlation might be due to the patient selection bias (all of recruited patients were adults with established lung disease as evidenced by an already low FEV_1_). Additional evaluation in an even larger group of CF patients, including children with milder lung disease, is warranted. We also observed that in the group of patients chronically infected with *P. aeruginosa*, 76.2% of the patients were dF508del homozygous as in the other group this was only 11.8%, confirming previous data concerning the effect of genotype on lung disease severity and pulmonary infection status [26].

The slightly higher expression of IL-17A mRNA expression in patients taking inhaled antibiotics could be explained by the fact that inhaled antibiotics are mostly taken by patients chronically infected with *P. aeruginosa*.

Patients taking AZI had similar levels of IL-17A and IL-23. We could not observe an effect of AZI on inflammatory cytokine expression in this cross-sectional study. Longitudinal controlled studies are required to study the effect of AZI on production of inflammatory cytokines.

The presence of IL-17A in sputum of CF patients outside a CF exacerbation indicates that IL-17A might be involved in the persistent neutrophilia present in the airways of CF patients. We did not find a correlation between IL-17A nor IL-23 sputum levels and sputum neutrophilia in stable CF patients. We acknowledge that measuring myeloperoxidase (MPO) or neutrophil elastase (NSE) might be a good alternative for the less reliable neutrophil sputum counts [27]. We found a significant reverse correlation between NSE and FEV_1 _(data not shown). It is yet to be investigated whether MPO or NSE are better markers for neutrophil inflammation to use in the clinical setting. NSE did however not correlate with IL-17A or IL-23 levels (data not shown). This suggests that other, IL-17-independent, pathways are also implicated in the neutrophil recruitment to the site of inflammation. C5a, LTB4 and platelet-activating factor (PAF) have been characterized as important neutrophil-stimulating mediators in CF airways [28]. Additional studies in an animal model will help us to determine whether blocking of IL-17A is able to reduce neutrophilic inflammation in CF airways.

IL-22 mRNA levels were very low or negative in our samples. This is in agreement with Aujla et al. [13], who found similarly low levels of IL-22 in BAL samples of CF patients during exacerbation, as opposed to high BAL levels for IL-17A and IL-23. As we have only recruited CF patients outside of an exacerbation, it seems logic that sputum IL-22 expression in these clinically stable patients would be even lower as observed in the study of Aujla et al. The observation that IL-22 was increased in lung tissue and lung lymphocytes of *P. aeruginosa*-infected patients with CF might suggest that IL-22 acts locally in the lung tissue, but is not produced into the airway lumen, in contrast to IL-17A.

Accidently, in our control group, only one male subject was present. Although theoretically it is always possible that gender difference might influence our results in the control population, we believe this will not bias our results. We indeed do not expect differences in cytokine mRNA expression depending on the gender in the control group since we also did not detect different levels of cytokines depending on the gender in the CF group.

As already mentioned, Th17 lymphocytes are considered to be the main source of IL-17A. However, lymphocyte percentages were very low in sputum of our CF patients. It seems thus unlikely that Th17 cells are the sole source of the expression of IL-17A in our samples. Other lymphocytes, like NKT cells [29] and γδ T cells [30] can also produce IL-17A. These lymphocytes however are even less abundant than CD4+ T cells and are unlikely to be the source of IL-17A found in the sputum samples of our patients. Immunostaining experiments showed that eosinophils express IL-17A mRNA in asthmatic patients [31]. In mice IL-17A mRNA expression was found in neutrophils [32] and macrophages [33]. The cellular source of the increased IL-17A in our CF patients remains to be elucidated. The cellular source of IL-23 in sputum is probably lung macrophages and dendritic cells.

## Conclusions

Our study is the first to show the presence of IL-17A protein and mRNA and IL-23 mRNA in sputum of a group of stable CF patients. These results suggest a potential role for the IL-23/IL-17A axis in CF lung inflammation and confirm that the adaptive immune system is involved in the pathophysiology of CF lung disease. Chronic infection with *P. aeruginosa *appears to be associated with higher mRNA expression of these cytokines in CF airways and additional longitudinal studies are needed to support the importance of IL-23/IL-17A mediated airway inflammation in the development of CF lung disease. This should allow us to determine the potential therapeutic value of blocking IL-17 in CF lung disease.

## Competing interests

The authors declare that they have no competing interests.

## Authors' contributions

AD carried out the sputum inductions, sputum processing, RT-PCR and CBA, drafted the manuscript. AWW performed RNA isolation, cDNA synthesis and RT-PCR of sputum samples and gave technical support. AK performed CBA analysis and helped with the problems encountered concerning protein measurements. KDB helped with study set-up and draft of the manuscript. DMB helped with set-up, technical support and first draft of the manuscript. LJD participated in the design and coordination of the study and helped to draft the manuscript. All authors read and approved the final manuscript.
